# MicroRNA-24 in Cancer: A Double Side Medal With Opposite Properties

**DOI:** 10.3389/fonc.2020.553714

**Published:** 2020-10-02

**Authors:** Sumei Wang, Nayan Liu, Qing Tang, Honghao Sheng, Shunqin Long, Wanyin Wu

**Affiliations:** ^1^Department of Oncology, Clinical and Basic Research Team of Traditional Chinese Medicine Prevention and Treatment of Non-Small Cell Lung Cancer, The Second Clinical College of Guangzhou University of Chinese Medicine, Guangzhou, China; ^2^Guangdong Provincial Hospital of Chinese Medicine, Guangzhou, China; ^3^Guangdong Provincial Key Laboratory of Clinical Research on Traditional Chinese Medicine Syndrome, Guangzhou, China; ^4^Guangdong Pharmaceutical University, Guangzhou, China

**Keywords:** miR-24, cancer, initiation, progression, biomarker

## Abstract

MicroRNA-24 (miR-24) has been widely studied in a variety of human cancers, which plays different roles in specific type of cancers. In the present review, we summarized the recent surveys regarding the role of miR-24 in different human cancers. On the one hand, miR-24 was reported to be down-regulated in some types of cancer, indicating its role as a tumor suppressor. On the other hand, it has shown that miR-24 was up-regulated in some other types of cancer, even in the same type of cancer, suggesting the role of miR-24 being as an oncogene. Firstly, miR-24 was dysregualted in human cancers, which is related to the clinical performance of cancer patients. Thus miR-24 could be used as a potential non-invasive diagnostic marker in human cancers. Secondly, miR-24 was associated with the tumor initiation and progression, being as a promoter or inhibitor. Therefore, miR-24 might be an effective prognostic biomarker in different type of cancers. Lastly, the abnormal expression of miR-24 was involved in the chemo- and radio- therapies of cancer patients, indicating the role of miR-24 being as a predictive biomarker to cancer treatment. Totally, miR-24 contributes to tumorigenesis, tumor progression, and tumor therapy, which closely related to clinic. The present review shows that miR-24 plays a double role in human cancers and provides plenty of evidences to apply miR-24 as a potential novel therapeutic target in treating human cancers.

## Introduction

MicroRNAs (miRNAs) are an evolutionarily conserved family of endogenous 19-22nt long non-coding RNAs, which are related with post-transcriptional regulation of gene expression by cleaving the target mRNAs or repressing translation (1). More and more evidences proved that miRNAs contributed to the initiation and progression of many diseases, including cancer. In the year 2002, the first relation between cancer and miRNA deregulation was demonstrated. Since then, a variety of surveys about miRNAs in different human cancers have been performed, showing miRNAs can be used as effective markers and as novel therapeutic targets in human cancers (2). For example, dysregulation of miRNAs has been widely involved in the activation of oncogenes in hepatocellular carcinoma (HCC), showing the potential diagnostic and therapeutic value of miRNAs in HCC (3). MiRNA dysregulation is a causal factor in many cancers, suppressing or promoting the initiation and progression of human cancers. Moreover, miRNA mimics and inhibitors that target miRNAs are promising in pre-clinical development, and several miRNA-targeted therapeutics have reached clinical development (4). Insights into the roles of miRNAs in human cancers have made miRNAs attractive tools and targets for novel therapeutic approaches.

According to many reports, microRNA-24 (miR-24) is associated with human cancer. The human miR-24 is located at chromosome 19 of the human genome and transcribed as a part of miR-23a-27a-24-2 cluster (5). Dysregulation of miR-24 has been reported in various human cancers, such as non-small cell lung cancer (NSCLC) (6), hepatocellular carcinoma (HCC) (7), breast cancer (BC) (8), nasopharyngeal carcinoma (NPC) (9), colorectal cancer (CRC) (10), laryngeal squamous cell carcinoma (LSCC) (11), and esophageal squamous cell carcinoma (ESCC) (12). In NSCLC, miR-24 functioned as an oncogene by targeting WWOX (WW domain-containing oxidoreductase), leading to the inhibition of NSCLC cell apoptosis by inactivating caspase-3 and the promotion of NSCLC cell growth and proliferation (13). Another study also reported that miR-24 was significantly up-regulated in NSCLC tissues compared with their corresponding non-tumorous tissues. And ectopic miR-24 expression promoted NSCLC cell migration and invasion by targeting ZNF367 (14). Compared with the paired normal tissues, miR-24 was increased in the hepatocellular carcinoma tissues (15). It was reported to promote HCC cell proliferation and migration and decrease cell apoptosis rates (16). In breast cancer, miR-24-3p was increased in patients with metastases, both in plasma and in patient tissues. And patients whose primary tumors expressed high levels of miR-24-3p showed a significantly lower survival rate compared to patients with low miR-24-3p levels in the TCGA (The Cancer Genome Atlas Program) cohort (17). MiR-24 was found to reduce breast cancer cell apoptosis, cleaved caspase-3 and the expression of p27 (18). In NPC, miR-24 was expressed significantly lower in NPC metastatic tumors, and its higher expression was associated with longer progression-free and metastasis-free survival durations. It could suppress NPC cell proliferation, invasion and migration via targeting c-Myc and then regulating epithelial-mesenchymal transition (EMT), indicating that miR-24 might be used as a prognostic factor and as a novel target for the prevention of NPC metastasis (19). In colorectal cancer, miR-24-1-5p could decrease cell proliferation and migration by repressing β-catenin expression, indicating its role as a tumor suppressor in CRC (20). However, another study showed that miR-24 was overexpressed in NK (natural killer) cells from CRC patients, compared with healthy volunteers. And overexpression of miR-24 suppressed secretions of IFN-γ (interferon-γ) and TNF-α (tumor necrosis factor-α) by targeting Paxillin, suggesting its role as an oncogene (21). This phenomenon might due to the different target of the same miRNA in the specific environment, even in the same cancer type. In laryngeal squamous cell carcinoma, miR-24 acted as an oncogene by promoting LSCC cell proliferation through regulating p27 (22). In esophageal squamous cell carcinoma, a previous study has reported that the level of serum miR-24 in ESCC patients is 4.82 times as high as that in healthy subjects (23). Recently, another study also showed that miR-24 was up-regulated in ESCC, reconfirming its role being as a prognostic biomarker for ESCC (12).

Several clinical evidences have supported the idea that dysregulation of miR-24 is correlated with the clinical features of human cancer. Therefore, the role of miR-24 in human cancer has been explored by numerous clinical, translational, and basic studies. The increasing amount of scientific evidence has confirmed the therapeutic relevance and the biological role of miR-24 in human cancer, for which a critical review is necessary. In the present review, we will summarize the recent advances in the research of miR-24 and provide an overview of its double roles in human cancers. We'll also comprehensively highlight the biological roles of miR-24 in carcinogenesis, cancer progression, metastasis, and drug resistance and discuss the potential application of miR-24 as a diagnostic tool and therapeutic target in human cancers.

## Therapeutic Relevance of miR-24 In Cancer

### Aberrant miR-24 Expression in Cancer

The expression pattern of miR-24 has been extensively studied, by comparing the tumor and non-tumor tissues of human cancer (Table 1). The up-regulation or down-regulation of miR-24 has been found in the specific cancer type. On the one hand, compared with normal tissues, miR-24 was found to be up-regulated in the following human cancers: lung cancer (LC) (6, 44, 45), hepatocellular carcinoma (HCC) (7), breast cancer (BC) (8), tongue squamous cell carcinoma (TSCC) (42), bladder cancer (BLC) (46), gastric cancer (GC) (47), acute leukemia (AL) (48), and Hodgkin Lymphoma (HL) (49). On the other hand, miR-24 was found to be down-regulated in the following human cancers: nasopharyngeal carcinoma (NPC) (9), colorectal cancer (CRC) (10), laryngeal squamous cell carcinoma (LSCC) (11), prostate cancer (PC) (27, 28), lung adenocarcinoma (LA) (29, 30), bladder cancer (BLC) (31), and gastric cancer (GC) (32), compared to the normal tissues. Specifically, miR-24 promoted lung cancer progression by regulating the tumor suppressor gene menin (45). While miR-24 was downregulated in both lung adenocarcinoma tissues and cells, and it suppressed the proliferation and migration of LA cells by regulating fibroblast growth factor receptor 3 (FGFR3) (30). Therefore, miR-24 was expressed differentially in different type of cancers, with opposite roles as an oncogene or a tumor suppressor.

**Table 1:** Dysregulation and mechanism of miR-24 in different types of human cancer.

**Cancer**	**Alteration**	**Mechanistically**	**References**
NPC	Down-regulated	Enhance radiosensitivity by targeting SP1, Jab1/CSN5, FSCN1	(9, 24–26)
CRC	Down-regulated	Decrease cell proliferation, migration	(10, 20)
LSCC	Down-regulated	Inhibit cell growth and enhance radiosensitivity by targeting XIAP	(11)
PC	Down-regulated	Increase cell apoptosis by targeting p27, p16, FSCN1	(27, 28)
LA	Down-regulated	Suppress cancer progression by targeting FGFR3, SOX18	(29, 30)
BLC	Down-regulated	Inhibit cell proliferation and metastasis by targeting CARMA3	(31)
GC	Down-regulated	Restrain cancer progression by downregulating RegIV	(32, 33)
PDA	Down-regulated	Inhibit cell proliferation by targeting LAMB3	(34)
RB	Down-regulated	Suppress cancer progression by targeting c-Myc	(35)
EC	Down-regulated	Suppress cell growth by targeting FERMT1	(36)
NSCLC	Up-regulated	Promote cancer progression	(6)
HCC	Up-regulated	Increase cell metastasis and invasion by targeting p53	(7, 15, 37)
BC	Up-regulated	Inhibit cell apoptosis by targeting Bim, ING5, p27Kip1, caspase-3	(8, 18, 38–41)
TSCC	Up-regulated	Promote cell growth, migration and invasion by targeting PTEN, FBXW7	(42, 43)
LC	Up-regulated	Promote cell growth and metastasis by targeting SOX7, menin	(44, 45)
BLC	Up-regulated	Promote cancer progression by inhibiting DEDD	(46)
GC	Up-regulated	Promote cell growth, migration; inhibit apoptosis by targeting BCL2L11	(47)
AL	Up-regulated	Promote cancer progression	(48)
HL	Up-regulated	Protect Hodgkin and Reed-Sternberg cells from apoptosis	(49)
OSCC	Up-regulated	Maintain cell proliferation through targeting PER1	(50)
LSCC	Up-regulated	Mediate the associations of titanium with chromosome damage and cancer	(51)
PAC	Up-regulated	Promote cell migration and invasion by targeting FZD5, TMEM92	(52)

*NPC, nasopharyngeal carcinoma; CRC, colorectal cancer; LSCC, laryngeal squamous cell carcinoma; PC, prostate cancer; LA, lung adenocarcinoma; BLC, bladder cancer; GC, gastric cancer; RB, retinoblastoma; PDA, pancreatic ductal adenocarcinoma; EC, esophageal cancer; NSCLC, non-small cell lung cancer; HCC, hepatocellular carcinoma; BC, breast cancer; TSCC, tongue squamous cell carcinoma; LC, lung cancer; AL, acute leukemia; HL, Hodgkin Lymphoma; OSCC, oral squamous cell carcinoma; LSCC, lung squamous cell carcinoma; PAC, pancreatic cancer*.

### Clinical Relevance of miR-24 in Cancer

A variety of findings have confirmed that dysregulation of miR-24 is related to the clinical performance of human cancer. Some studies found that the expression of miR-24 in cancer tissues, being as an oncogene, was correlated with a later clinical stage, as well as the extent of metastasis (53). For example, miR-24-3p was discovered to be down-regulated in CRC tissues compared with their corresponding non-cancerous tissues, and its expression was associated with local invasion (*P* = 0.002), lymph node metastasis (*P* = 0.0007) and clinical stage (*P* < 0.001) significantly (10). Another report found that the ratio of miR-21/24 was significantly correlated with the tumor size, TNM (tumor, lymph node, and metastasis) stage, lymph metastasis and histologic differentiation of CRC (54). The above two studies showed that miR-24 could be used as a potential prognostic biomarker and survival risk factor for CRC patients. MiR-24 was found to be significantly associated with survival of patients with glioblastoma (GBM) multiforme, and promoted GBM progression by targeting tumor suppressor sex-determining region Y-box 7 (SOX7) (55, 56). MiR-24 was obviously overexpressed in lung carcinoma tissues than that of para-cancerous tissues. And the overall survival (OS) of patients with higher miR-24 expression was remarkably shorter than those with lower expression. They further proved that miR-24 promoted the viability, proliferation and cell cycle of lung carcinoma cells and inhibited cell apoptosis by binding to MAPK7 (mitogen-activated protein kinase 7) (57). In tongue squamous cell carcinoma, a double role of miR-24 was discovered relating to its role in clinic. A study reported that the decrease of miR-24 expression was correlated with high grade and late stage tumor in TSCC (42). Nevertheless, another study reported that miR-24 was increased in both TSCC tissues and cell lines, and the increase of miR-24 expression was associated with advanced clinical stage and a shorter overall survival of TSCC patients (43). In total, these evidences suggest the significance of miR-24, in terms of its correlation with the staging and survival of patients with cancer, being as a potential prognostic biomarker for human cancer.

### miR-24 as a Diagnostic and Prognostic Marker in Cancer

The differential expression of miR-24 in body fluid such as plasma, alone or in combination with a panel of other miRNAs, makes it might be considered as a potential non-invasive marker in cancer diagnosis. Since miRNAs are related to the clinic, detecting and monitoring the dysregulated miRNAs could reveal the stage of cancer. There are many evidences revealed the diagnostic role of miR-24 in cancers (58). Meng et al. (59) found that serum miR-24-3p could discriminate HCC patients from chronic liver disease (CLD), with an AUC (area under the ROC curve) of 0.636 [95% confidence interval (CI) 0.524–0.748]. And the combination of serum miR-24-3p and AFP (alpha fetoprotein) could improve the diagnostic accuracy for HCC, compared to each biomarker alone. Zhu et al. (33) reported that the differentially expressed circulatory plasma miR-24-3p together with miR-425-5p, miR-1180-3p, miR-122-5p, and miR-4632-5p could be considered as a novel potential biomarker panel for the diagnosis of early gastric cancer. Fang et al. (60) identified a panel of plasma miRNAs, including miR-24, could be a helpful diagnostic marker for colorectal carcinoma detection, especially for its early stage. This is superior to the currently used clinical biomarkers for CRC detection, such as carcino-embryonic antigen (CEA) and carbohydrate antigen19-9 (CA19-9) (60). Fredsoe et al. (61) validated a previously identified 3-miRNA diagnostic ratio model, (miR-222-3p^*^miR-24-3p/miR-30c-5p) for prostate cancer in cell-free urine. A panel of plasma miRNAs, including miR-24, was validated as a diagnostic biomarker for childhood acute lymphoblastic leukemia detection (62). A study found that miR-24-3p had excellent diagnostic accuracy for oral squamous cell carcinoma [(AUC) = 0.738; *P* = 0.02], thus salivary exosomal miR-24-3p could be a potential novel diagnostic biomarker for OSCC (50). In total, the above evidences confirmed the role of miR-24 being as a diagnostic marker in human cancer.

Regarding the differential expression of miR-24 in human cancer, miR-24 is associated with patient survival, indicating it could be a potential prognostic biomarker in cancer. Franchina et al. (6) found that circulating miRNAs including miR-22, miR-24, and miR-34a could function as novel predictive biomarkers to pemetrexed-based chemotherapy in advanced non-small cell lung cancer. Researchers discovered that the elevation of serum miR-24-3p could be an independent poor prognostic factor for OS and DFS (disease free survival) of HBV (hepatitis B virus)-related HCC patients (59). And in acute leukemia, Kaplan-Meier analysis showed that AL patients with high miR-24 expression tended to have shorter overall survival, and in the multivariate analysis stratified for known prognostic variables, miR-24 was identified as an independent prognostic marker (48). In contrast, CRC patients with low miR-24-3p level had a significantly poorer prognosis than those with high miR-24-3p level. And multivariate analysis revealed that miR-24-3p could be an independent prognostic indicator for OS of CRC patients (10). In breast cancer, miR-24 was identified to be highly predictive of early breast cancer relapse (38). In nasopharyngeal carcinoma, a survey proved that exosomal miR-24-3p could serve as a prognostic biomarker, due to its involvement in tumor pathogenesis by mediating T-cell inhibition (25). And our previous study validated that miR-24 could serve as a prognostic marker for NPC recurrence (24). Therefore, miR-24 is a potential promising diagnostic and prognostic marker in human cancer.

## Biological Role of The miR-24 In Human Cancer

### Carcinogenesis and Progression

**Figure 1:**
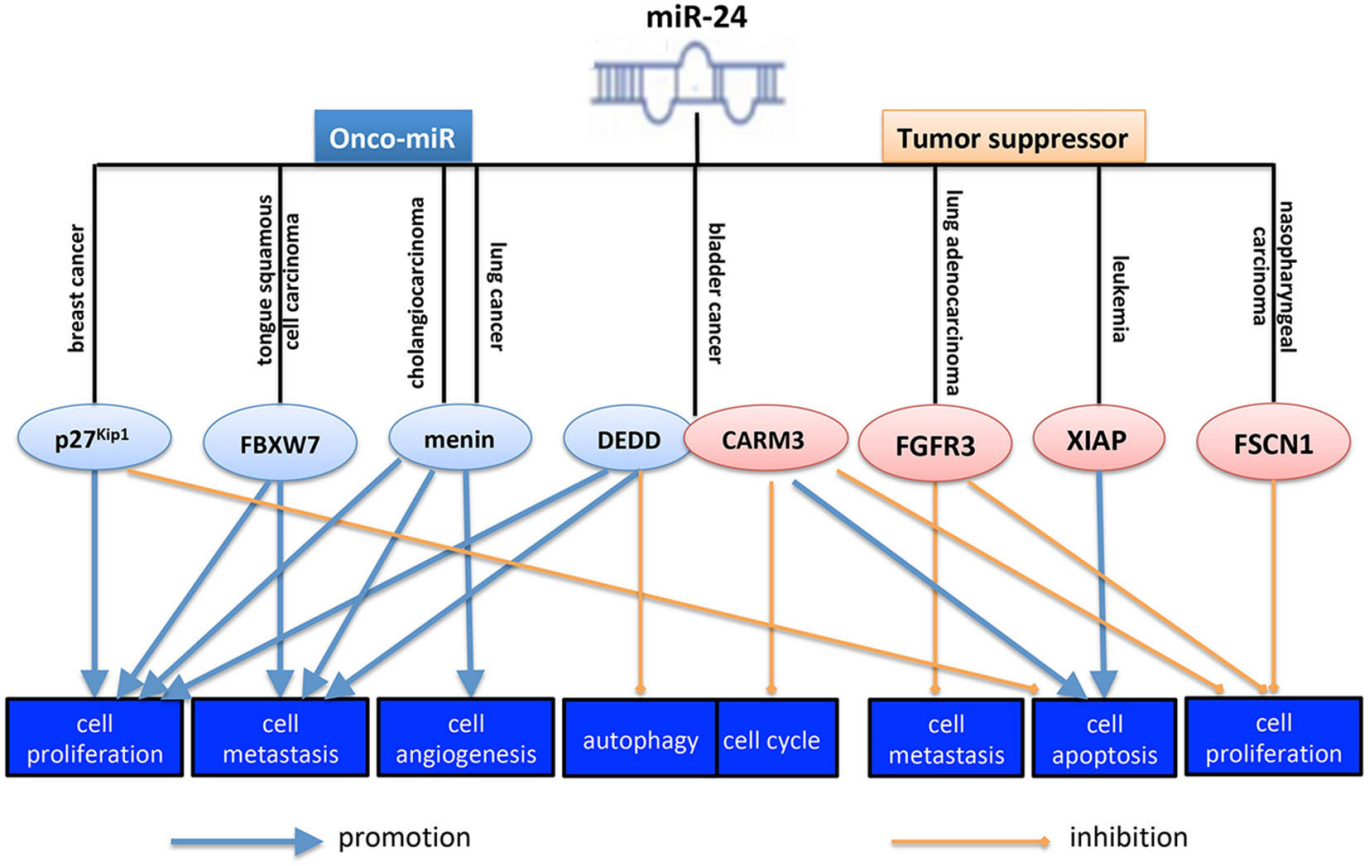
Role of miR-24 in cancer initiation and progression. MiR-24 functioned as a tumor suppressor or an onco-miR, in different cancer types, even in the same cancer type, by targeting different targets. By targeting specific targets, miR-24 could suppress or inhibit cancer cell proliferation, metastasis, angiogenesis, autophagy, cell cycle, and cell apoptosis. The pink ellipse means the protein in it functions as an onco-protein, and the blue ellipse means the protein in it functions as a tumor suppressor. p27^Kip1^, a key protein in regulating cell cycle; FBXW7, F-box and WD repeat domain containing 7, a substrate-recognition subunit of a ubiquitin ligase complex; menin, the protein product of the *MEN1* gene, a ubiquitously expressed protein that lacks homology with other protein families; DEDD, Asp-Glu-Asp-Asp domain, a death effector domain-containing protein; CARM3, CARD-containing MAGUK protein 3, a scaffold protein known to activate NF-κB pathway; FGFR3, fibroblast growth factor receptor 3; XIAP, X-linked inhibitor of apoptosis protein; FSCN1, fascin actin-bundling protein 1, a protein encoded by an actin-bundling protein, FSCN1, which is involved in formation of actin-based structures that contribute to cell migration.

miR-24 is involved in the initiation and progression of human cancer by regulating the specific gene. On the one hand, miR-24 promotes tumorigenesis in some cancer types. Zhang et al. (63) reported that miR24-1-5p promoted tumorigenesis in ovarian epithelial cells. And hsa-mir-24-2 has been validated to be associated with the metastasis of cervical squamous cell carcinoma (64). In lung squamous cell carcinoma, miR-24-3p was overexpressed and functioned as an onco-miR (51). In mesothelioma, miR-24-3p promoted tumorigenesis by inducing cancer cell growth and regulating Rho-GTP activity positively (65). In hepatocellular carcinoma, miR-24-3p increased cancer cell viability and reduced its cell apoptosis (15). And miR-24 increased cell metastasis and invasion by targeting p53 (7). In lung cancer, miR-24 promoted cancer cell growth and metastasis and inhibited cell apoptosis also by targeting menin, and SOX7 (sex-determining region Y-box 7) (44, 45). In colorectal cancer, a study showed that HIF-1α (hypoxia inducible factor-1α)-induced miR-23a~27a~24 cluster could promote cancer progression via reprogramming its metabolism (66), suggesting miR-24 might be an onco-miR in CRC. Although a previous study showed that miR-24-3p functioned as a tumor suppressor in CRC (10). Whether miR-24 functioned as a tumor suppressor or an onco-miR, in different cancer types, or even in the same cancer type, depends on its different targets. By targeting specific targets, miR-24 could suppress or promote cancer cell proliferation, metastasis, angiogenesis, autophagy, cell cycle, and cell apoptosis. MiR-24-3p mediated the tumorigenesis promotion and accelerated xenografted tumor growth of breast cancer (39, 40). As shown in Figure 1, overexpressing miR-24-3p promoted cell proliferation and inhibited cell apoptosis in breast cancer by targeting p27^Kip1^ (8, 18). In tongue squamous cell carcinoma, miR-24 promoted its proliferation, migration and invasion through targeting FBXW7 (F-box and WD repeat domain containing 7) (43). In cholangiocarcinoma, miR-24 increased cancer cell proliferation, angiogenesis, migration, and invasion by regulating menin (67). In bladder cancer, miR-24-3p increased cell proliferation and migration ability by targeting DEDD (Asp-Glu-Asp-Asp domain) (46).

On the other hand, however, miR-24 suppresses tumorigenesis in some human cancers. For example, miR-24 was significantly downregulated in gastric cancer tissues compared with matched non-tumor tissues, and it was associated with tumor differentiation (32). In CRC, miR-24-3p suppressed cancer cell proliferation, cell migration and invasion, functioning as a tumor suppressor (10). And miR-24-1-5p decreased CRC cell proliferation, migration and survival significantly by repressing β-catenin expression (20). Ectopic expression of miR-24 inhibited cell cycle, proliferation, migration, and clonogenic potential of prostate cancer cells, as well as inducing cell apoptosis (27). In pancreatic ductal adenocarcinoma (PDA), miR-24-3p exerted its anti-cancer role by suppressing the expression of Laminin Subunit Beta 3 (LAMB3), an oncogene (34). In retinoblastoma, miR-24 plays a tumor suppressive role by targeting c-Myc (35). Moreover, even in the same type of cancer, miR-24 functions oppositely. For example, in colorectal cancer, overexpression of miR-24-1-5p significantly repressed β-catenin expression, and simultaneously decreased CRC cell migration (20). Michael et al. (68) also confirmed that miR-24 inhibited the cell growth of both lung and colon carcinoma. As shown in Figure 1, in bladder cancer, another study reported that miR-24 inhibited cell proliferation, arrested cell cycle and induced cell apoptosis by targeting CARMA3 (CARD-containing MAGUK protein 3) (31). And In lung adenocarcinoma, miR-24-3p could suppress cell proliferation, migration, and invasion by regulating FGFR3 directly (30). In acute lymphoblastic leukemia, miR-24-3p induced cell apoptosis by regulating XIAP (X-linked inhibitor of apoptosis protein) (69). In NPC, miR-24 suppressed cell prolifearation, migration, and invasion and increased the readiosensitivity of cells to iridiation by targeting SP1 (specificity protein 1), FSCN1 (fascin actin-bundling protein 1), and Jab1 (Jun activation domain-binding protein 1), respectively, funcioning as a tumor suppressor (9, 24, 26). Therefore, both positive and negative roles of the miR-24, in cancer cell maintenance, were found in different kinds of cancer, even in the same cancer type (Figure 1).

### Cancer Therapy Resistance

**Figure 2:**
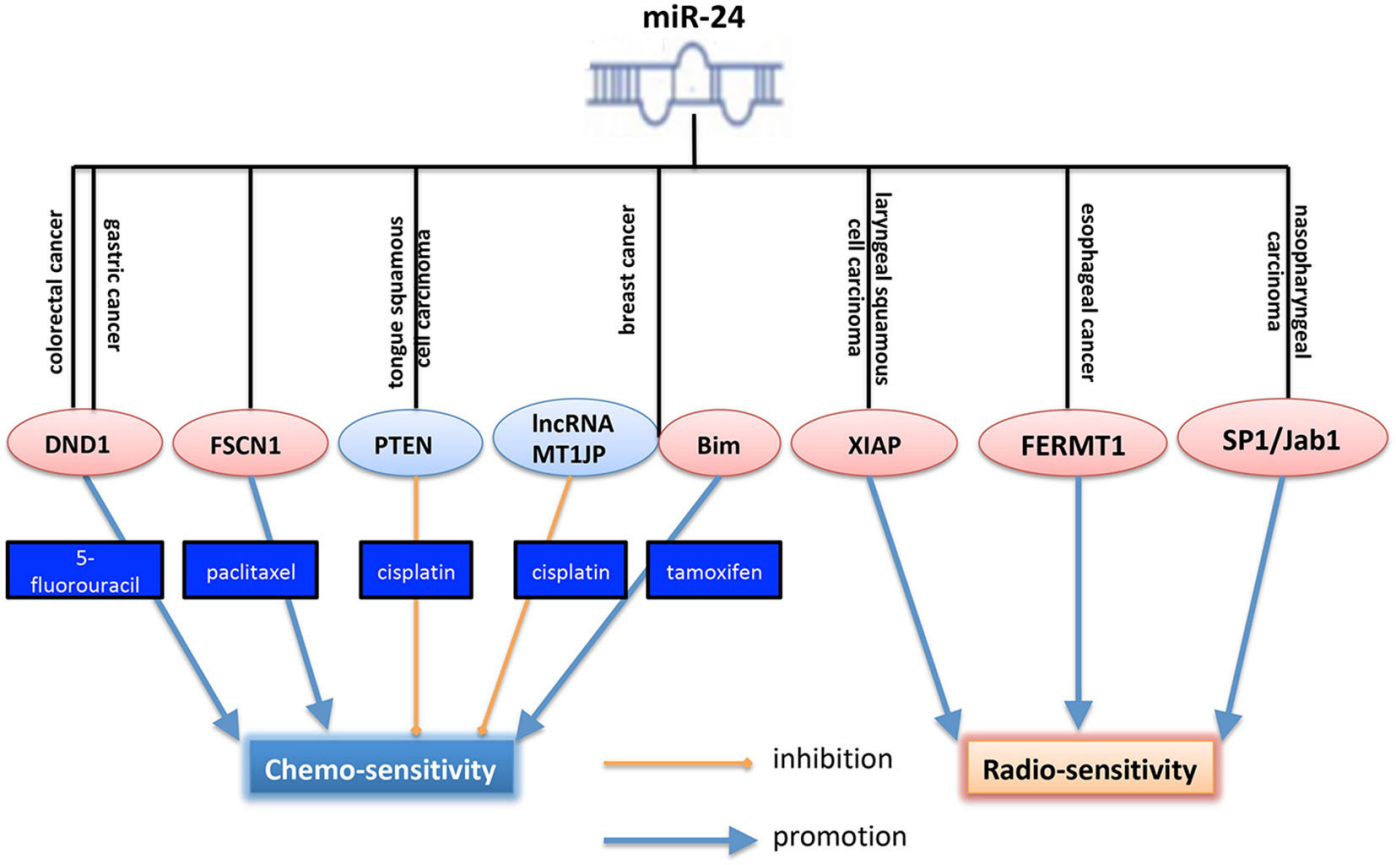
Role of miR-24 in cancer chemo- and radio- therapies. MiR-24 increased 5-FU sensitivity in both colorectal and gastric cancers. However, miR-24 decreased cisplatin sensitivity in both TSCC and breast cancer. Especially, in breast cancer, miR-24 enhanced tamoxifen senstitivy by targeting Bim. And miR-24 increased sensitivity of cancer cells to radiotherapy, and showed a dual role in chemotherapy. By targeting different targets, miR-24 increased radio-sensitivity in NPC, EC and LSCC. The pink ellipse means the protein in it functions as an onco-protein, and the blue ellipse means the protein in it functions as a tumor suppressor. DND1, dead end protein 1, a RNA-binding protein; FSCN1, fascin actin-bundling protein 1, a protein encoded by an actin-bundling protein, FSCN1, which is involved in formation of actin-based structures that contribute to cell migration. PTEN, phosphatase and tensin homolog, a well-known inhibitor of PI3K/Akt pathway; XIAP, X-linked inhibitor of apoptosis protein; FERMT1, also known as KINDLIN-1, is a FERM domain containing adaptor protein that is found predominantly at cell-extracellular matrix adhesions where it binds to integrin β subunits and is required for efficient integrin activation; SP1, specificity protein 1; Jab1, Jun activation domain-binding protein 1, an onco-protein.

MiR-24 not only plays a crucial in tumor initiation and progression, but also plays an essential role in cancer therapy, including chemo- and radio- therapies. Drug resistance limits the efficacy of chemotherapy in human cancers. For the role of miR-24 in cancer chemotherapy, a lot of researchers provided evidences. As shown in Figure 2, in colorectal cancer, some reports showed that miR-24 functioned as a tumor suppressor. It was downregulated in colorectal cancer cells and induced cell apoptosis. What's more, ectopic expression of miR-24 could enhance the chemosensitivity of CRC cells to 5-fluorouracil (5-FU) by targeting RNA-binding protein DND1 (dead end protein 1) (70). In gastric cancer, DND1 was a target of miR-24. And miR-24 overexpression suppressed the migration and invasion of GC. It also enhanced the chemosensitivity of the SNU1 gastric cancer cells (71). In prostate cancer, overexpression of miR-24-3p inhibited survival rate, half maximal inhibitory concentration (IC50) of paclitaxel (PTX) but increased apoptosis in prostate cancer cells after treatment of PTX, via regulating fascin1 (FSCN1) (28). Those researches showed that miR-24 functioned differently after the treatment with chemotherapy, might due to the difference of the specific microenvironment. In human tongue squamous cell carcinoma, miR-24 increased cisplatin resistance by targeting PTEN (phosphatase and tensin homolog) and then acrivating Akt pathway (42). In breast cancer, miR-24-3p functioned as an onco-miR and decreased cisplatin sensitivity by negatively regulating lncRNA (long non-coding RNA) MT1JP (39). Interestingly, another study, published at the same year for the same cancer type, reported that miR-24-3p increased tamoxifen sensitivity by targeting Bim, leading to the induction of breast cancer cell apoptosis, acting as a tumor suppressor (41).

For the role of miR-24 in radiotherapy, Xu et al. (11) showed that miR-24 could increase LSCC radio-sensitivity through enhancing irradiation-induced apoptosis. As shown in Figure 2, in laryngeal squamous cell carcinoma, miR-24 was identified to suppress tumor growth, induce cell apoptosis, and reverse cancer cell radioresistance via targeting XIAP (11). In esophageal cancer, miR-24 could also increase its radio-sensitivity by suppressing FERMT1 (also known as KINDLIN-1) (36). Kang et al. (9) and we previously reported that miR-24 enhanced radio-sensitivity of NPC by targeting SP1, and Jab1 (9, 24). Totally, miR-24 plays a crucial role in the radio- and chemo- therapies of cancer (Figure 2).

## Discussion

Non-coding RNAs (ncRNAs) are important regulators of gene expression in both physiological and pathological conditions, which is critical to finalize the pharmacological application of ncRNAs as either therapeutic tools or as molecular biomarkers in human cancer (72). In the present review, we summarized that miR-24 had opposite functions, even in the same type of cancer (32, 47). Firstly, this could be probably due to the inconsistent quality of the studies. Therefore, further large studies are needed to confirm these results, and quality control should be highlighted in future investigations of miR-24 in order to produce consistent and reliable conclusions. Secondly, this could be probably due to the complex regulation network of miR-24. Though miR-24 has promising diagnostic and prognostic value for a variety of human cancers, and its roles in cancer progression suggest that it can be a useful biomarker for therapy, its clinical application remains rare. This is mainly because its toxicity and safety remain unclear. Therefore, further studies are needed to confirm its functions and adverse effects.

## Conclusions

This review highlighted the scientific achievements in the study of miR-24 in human cancer and outlined the biological and clinical insights on the advancements. MiR-24 has been detected as being differentially expressed across different types of cancer, and might predict the survival and treatment response of cancer patients. Its biological functions covered the scenarios of carcinogenesis, cancer progression, metastasis, and drug resistance, suggesting that it might have potential to be used as an emerging targetable entity in cancer treatment. Throughout the present review, we conclude that “microRNA-24 in cancer: a double side medal with opposite properties.”

## Author Contributions

SW was responsible for writing and editing the manuscript. NL was responsible for collecting references and writing the manuscript. QT and HS collected some references. SL and WW were responsible for modifying the manuscript. All authors contributed to the article and approved the submitted version.

## Conflict of Interest

The authors declare that the research was conducted in the absence of any commercial or financial relationships that could be construed as a potential conflict of interest.

## Abbreviations

miR-24: microRNA-24
miRNAs: microRNAs
HCC: hepatocellular carcinoma
NSCLC: non-small cell lung cancer
BC: breast cancer
NPC: nasopharyngeal carcinoma
CRC: colorectal cancer
LSCC: laryngeal squamous cell carcinoma
ESCC: esophageal squamous cell carcinoma
WWOX: WW domain-containing oxidoreductase
TCGA: The Cancer Genome Atlas Program
EMT: epithelial-mesenchymal transition
NK: natural killer cell
IFN-γ: interferon-γ
TNF-α: tumor necrosis factor-α
LC: lung cancer
TSCC: tongue squamous cell carcinoma
BLC: bladder cancer
GC: gastric cancer
AL: acute leukemia
HL: Hodgkin Lymphoma
PC: prostate cancer
LA: lung adenocarcinoma
FGFR3: fibroblast growth factor receptor 3
OSCC: oral squamous cell carcinoma
LSCC: lung squamous cell carcinoma
PAC: pancreatic cancer
PDA: pancreatic ductal adenocarcinoma
RB: retinoblastoma
EC: esophageal cancer
TNM: tumor, lymph node, and metastasis
GBM: glioblastoma
OS: overall survival
MAPK7: mitogen-activated protein kinase 7
CLD: chronic liver disease
AUC: area under the ROC curve
CI: confidence interval
AFP: alpha fetoprotein
CEA: carcino-embryonic antigen
CA-199: carbohydrate antigen19-9
DFS: disease free survival
HBV: hepatitis B virus
DEDD: Asp-Glu-Asp-Asp domain
FBXW7: F-box and WD repeat domain containing 7
SOX7: sex-determining region Y-box 7
HIF-1α: hypoxia inducible factor-1α
XIAP: X-linked inhibitor of apoptosis protein
LAMB3: Laminin Subunit Beta 3
SP1: specificity protein 1
FSCN1: fascin actin-bundling protein 1
Jab1: Jun activation domain-binding protein 1
CARMA3: CARD-containing MAGUK protein 3
FERMT1: also known as KINDLIN-1
PTEN: phosphatase and tensin homolog
lncRNA: long non-coding RNA
5-FU: 5-fluorouracil
DND1: dead end protein 1
IC50: half maximal inhibitory concentration
PTX: paclitaxel
ncRNAs: non-coding RNAs.

## References

[1] Bartel DP. MicroRNAs: genomics, biogenesis, mechanism, and function. Cell. (2004) 116:281–97. 10.1016/S0092-8674(04)00045-5 14744438

[2] Acunzo M Romano G Wernicke D Croce CM. MicroRNA and cancer–a brief overview. Adv Biol Regul. (2015) 57:1–9. 10.1016/j.jbior.2014.09.013 25294678

[3] Wong C-M Tsang FH-C Ng IO-L. Non-coding RNAs in hepatocellular carcinoma: molecular functions and pathological implications. Nat Rev Gastroenterol Hepatol. (2018) 15:137–51. 10.1038/nrgastro.2017.169 29317776

[4] Rupaimoole R Slack FJ. MicroRNA therapeutics: towards a new era for the management of cancer and other diseases. Nat Rev Drug Discov. (2017) 16:203–22. 10.1038/nrd.2016.246 28209991

[5] Chhabra R Dubey R Saini N. Cooperative and individualistic functions of the microRNAs in the miR-23a~27a~24-2 cluster and its implication in human diseases. Mol Cancer. (2010) 9:232. 10.1186/1476-4598-9-232 20815877 PMC2940846

[6] Franchina T Amodeo V Bronte G Savio G Ricciardi GR Picciotto M. Circulating miR-22, miR-24 and miR-34a as novel predictive biomarkers to pemetrexed-based chemotherapy in advanced non-small cell lung cancer. J Cell Physiol. (2014) 229:97–9. 10.1002/jcp.24422 23794259

[7] Chen L Luo L Chen W Xu HX Chen F Chen LZ. MicroRNA-24 increases hepatocellular carcinoma cell metastasis and invasion by targeting p53: miR-24 targeted p53. Biomed Pharmacother. (2016) 84:1113–8. 10.1016/j.biopha.2016.10.051 27780140

[8] Lu K Wang J Song Y Zhao S Liu H Tang D. miRNA-24-3p promotes cell proliferation and inhibits apoptosis in human breast cancer by targeting p27Kip1. Oncol Rep. (2015) 34:995–1002. 10.3892/or.2015.4025 26044523

[9] Kang M Xiao J Wang J Zhou P Wei T Zhao T. MiR-24 enhances radiosensitivity in nasopharyngeal carcinoma by targeting SP1. Cancer Med. (2016) 5:1163–73. 10.1002/cam4.660 26922862 PMC4924375

[10] Gao Y Liu Y Du L Li J Qu A Zhang X. Down-regulation of miR-24-3p in colorectal cancer is associated with malignant behavior. Med Oncol. (2015) 32:362. 10.1007/s12032-014-0362-4 25502080

[11] Xu L Chen Z Xue F Chen W Ma R Cheng S. MicroRNA-24 inhibits growth, induces apoptosis, and reverses radioresistance in laryngeal squamous cell carcinoma by targeting X-linked inhibitor of apoptosis protein. Cancer Cell Int. (2015) 15:61. 10.1186/s12935-015-0217-x 26106283 PMC4477309

[12] Maghsudlu M Farashahi Yazd E Amiriani T. Increased expression of MiR-27a and MiR-24-2 in esophageal squamous cell carcinoma. J Gastrointest Cancer. (2020) 51:227–33. 10.1007/s12029-019-00232-x 31020570

[13] Wang XH Gan CZ Xie JY. Inhibition of miR-24 suppresses malignancy of human non-small cell lung cancer cells by targeting WWOX *in vitro* and *in vivo*. Thorac Cancer. (2018) 9:1583–93. 10.1111/1759-7714.12824 30307120 PMC6275841

[14] Liu Z Jiang L Zhang G Li S Jiang X. MiR-24 promotes migration and invasion of non-small cell lung cancer by targeting ZNF367. J BUON. (2018) 23:1413–9. 30570867

[15] Fan JC Zeng F Le YG Xin L. LncRNA CASC2 inhibited the viability and induced the apoptosis of hepatocellular carcinoma cells through regulating miR-24-3p. J Cell Biochem. (2018) 119:6391–7. 10.1002/jcb.26479 29091305

[16] Shan QL Chen NN Meng GZ Qu F. Overexpression of lncRNA MT1JP mediates apoptosis and migration of hepatocellular carcinoma cells by regulating miR-24-3p. Cancer Manag Res. (2020) 12:4715–24. 10.2147/CMAR.S249582 32606962 PMC7308148

[17] Khodadadi-Jamayran A Akgol-Oksuz B Afanasyeva Y Heguy A Thompson M Ray K. Prognostic role of elevated mir-24-3p in breast cancer and its association with the metastatic process. Oncotarget. (2018) 9:12868–78. 10.18632/oncotarget.24403 29560116 PMC5849180

[18] Yu B Gao W Zhou H Miao X Chang Y Wang L. Propofol induces apoptosis of breast cancer cells by downregulation of miR-24 signal pathway. Cancer Biomark. (2018) 21:513–9. 10.3233/CBM-170234 29103019 PMC13078298

[19] Su B Xu T Bruce JP Yip KW Zhang N Huang Z. hsamiR24 suppresses metastasis in nasopharyngeal carcinoma by regulating the cMyc/epithelialmesenchymal transition axis. Oncol Rep. (2018) 40:2536–46. 10.3892/or.2018.6690 30226609 PMC6151896

[20] Zhang H Guo J Mao L Li Q Guo M Mu T. Up-regulation of miR-24-1-5p is involved in the chemoprevention of colorectal cancer by black raspberry anthocyanins. Br J Nutr. (2019) 122:518–526. 10.1017/S0007114518003136 30375302

[21] Zhang LL Zhang LF Shi YB. miR-24 inhibited the killing effect of natural killer cells to colorectal cancer cells by downregulating Paxillin. Biomed Pharmacother. (2018) 101:257–63. 10.1016/j.biopha.2018.02.024 29494963

[22] Shen Z Hao W Zhou C Deng H Ye D Li Q. Long non-coding RNA AC026166.2-001 inhibits cell proliferation and migration in laryngeal squamous cell carcinoma by regulating the miR-24-3p/p27 axis. Sci Rep. (2018) 8:3375. 10.1038/s41598-018-21659-5 29463827 PMC5820272

[23] Dong W Li B Wang Z Zhang Z Wang J. Clinical significance of microRNA-24 expression in esophageal squamous cell carcinoma. Neoplasma. (2015) 62:250–8. 10.4149/neo_2015_030 25591590

[24] Wang S Pan Y Zhang R Xu T Wu W Zhang R. Hsa-miR-24-3p increases nasopharyngeal carcinoma radiosensitivity by targeting both the 3'UTR and 5'UTR of Jab1/CSN5. Oncogene. (2016) 35:6096–108. 10.1038/onc.2016.147 27157611 PMC5102828

[25] Ye SB Zhang H Cai TT Liu YN Ni JJ He J. Exosomal miR-24-3p impedes T-cell function by targeting FGF11 and serves as a potential prognostic biomarker for nasopharyngeal carcinoma. J Pathol. (2016) 240:329–40. 10.1002/path.4781 27538493

[26] Li YQ Lu JH Bao XM Wang XF Wu JH Hong WQ. MiR-24 functions as a tumor suppressor in nasopharyngeal carcinoma through targeting FSCN1. J Exp Clin Cancer Res. (2015) 34:130. 10.1186/s13046-015-0242-6 26503504 PMC4621856

[27] Lynch SM McKenna MM Walsh CP McKenna DJ. miR-24 regulates CDKN1B/p27 expression in prostate cancer. Prostate. (2016) 76:637–48. 10.1002/pros.23156 26847530

[28] Li X Han X Wei P Yang J Sun J. Knockdown of lncRNA CCAT1 enhances sensitivity of paclitaxel in prostate cancer via regulating miR-24-3p and FSCN1. Cancer Biol Ther. (2020) 21:452–62. 10.1080/15384047.2020.1727700 32089062 PMC7515504

[29] Olbromski M Rzechonek A Grzegrzolka J Glatzel-Plucinska N Chachaj A Werynska B. Influence of miR-7a and miR-24-3p on the SOX18 transcript in lung adenocarcinoma. Oncol Rep. (2018) 39:201–8. 10.3892/or.2017.6077 29115529

[30] Jing P Zhao N Xie N Ye M Zhang Y Zhang Z. miR-24-3p/FGFR3 signaling as a novel axis is involved in epithelial-mesenchymal transition and regulates lung adenocarcinoma progression. J Immunol Res. (2018) 2018:2834109. 10.1155/2018/2834109 29850625 PMC5933034

[31] Zhang S Zhang C Liu W Zheng W Zhang Y Wang S. MicroRNA-24 upregulation inhibits proliferation, metastasis and induces apoptosis in bladder cancer cells by targeting CARMA3. Int J Oncol. (2015) 47:1351–60. 10.3892/ijo.2015.3117 26252200

[32] Duan Y Hu L Liu B Yu B Li J Yan M. Tumor suppressor miR-24 restrains gastric cancer progression by downregulating RegIV. Mol Cancer. (2014) 13:127. 10.1186/1476-4598-13-127 24886316 PMC4041902

[33] Zhu XL Ren LF Wang HP Bai ZT Zhang L Meng WB. Plasma microRNAs as potential new biomarkers for early detection of early gastric cancer. World J Gastroenterol. (2019) 25:1580–91. 10.3748/wjg.v25.i13.1580 30983818 PMC6452233

[34] Huang W Gu J Tao T Zhang J Wang H Fan Y. MiR-24-3p inhibits the progression of pancreatic ductal adenocarcinoma through LAMB3 downregulation. Front Oncol. (2019) 9:1499. 10.3389/fonc.2019.01499 32039003 PMC6985431

[35] Yu F Pang G Zhao G. ANRIL acts as onco-lncRNA by regulation of microRNA-24/c-Myc, MEK/ERK and Wnt/beta-catenin pathway in retinoblastoma. Int J Biol Macromol. (2019) 128:583–92. 10.1016/j.ijbiomac.2019.01.157 30703428

[36] Yan Q Chen T Yang H Yu H Zheng Y He T. The effect of FERMT1 regulated by miR-24 on the growth and radiation resistance of esophageal cancer. J Biomed Nanotechnol. (2019) 15:621–31. 10.1166/jbn.2019.2711 31165706

[37] Jin X Cai L Wang C Deng X Yi S Lei Z. CASC2/miR-24/miR-221 modulates the TRAIL resistance of hepatocellular carcinoma cell through caspase-8/caspase-3. Cell Death Dis. (2018) 9:318. 10.1038/s41419-018-0350-2 29476051 PMC5833678

[38] Sochor M Basova P Pesta M Bartos J Stopka T. Prediction potential of serum miR-155 and miR-24 for relapsing early breast cancer. Eur J Cancer. (2018) 92:S121. 10.1016/S0959-8049(18)30585-9 28994735 PMC5666798

[39] Zhu D Zhang X Lin Y Liang S Song Z Dong C. MT1JP inhibits tumorigenesis and enhances cisplatin sensitivity of breast cancer cells through competitively binding to miR-24-3p. Am J Transl Res. (2019) 11:245–56. 30787983 PMC6357327

[40] Cui S Liao X Ye C Yin X Liu M Hong Y. ING5 suppresses breast cancer progression and is regulated by miR-24. Mol Cancer. (2017) 16:89. 10.1186/s12943-017-0658-z 28490335 PMC5424299

[41] Han X Li Q Liu C Wang C Li Y. Overexpression miR-24-3p repressed Bim expression to confer tamoxifen resistance in breast cancer. J Cell Biochem. (2019) 120:12966–76. 10.1002/jcb.28568 31001849

[42] Zheng X Li J Peng C Zhao J Chi J Meng X. MicroRNA-24 induces cisplatin resistance by targeting PTEN in human tongue squamous cell carcinoma. Oral Oncol. (2015) 51:998–1003. 10.1016/j.oraloncology.2015.08.002 26365986

[43] Zhao J Hu C Chi J Li J Peng C Yun X. miR-24 promotes the proliferation, migration and invasion in human tongue squamous cell carcinoma by targeting FBXW7. Oncol Rep. (2016) 36:1143–9. 10.3892/or.2016.4891 27350307

[44] Yan L Ma JZ Zhu YP Zan JW Wang Z Ling LF. miR-24-3p promotes cell migration and proliferation in lung cancer by targeting SOX7. J Cell Biochem. (2018) 119:3989–98. 10.1002/jcb.26553 29231262

[45] Pan Y Wang H Ma D Ji Z Luo L Cao F. miR24 may be a negative regulator of menin in lung cancer. Oncol Rep. (2018) 39:2342–50. 10.3892/or.2018.6327 29565463

[46] Yu G Jia Z Dou Z. miR-24-3p regulates bladder cancer cell proliferation, migration, invasion and autophagy by targeting DEDD. Oncol Rep. (2017) 37:1123–31. 10.3892/or.2016.5326 28000900

[47] Zhang H Duan J Qu Y Deng T Liu R Zhang L. Onco-miR-24 regulates cell growth and apoptosis by targeting BCL2L11 in gastric cancer. Protein Cell. (2016) 7:141–51. 10.1007/s13238-015-0234-5 26758252 PMC4742383

[48] Organista-Nava J Gomez-Gomez Y Illades-Aguiar B Del Carmen Alarcon-Romero L Saavedra-Herrera MV Rivera-Ramirez AB. High miR-24 expression is associated with risk of relapse and poor survival in acute leukemia. Oncol Rep. (2015) 33:1639–49. 10.3892/or.2015.3787 25672522 PMC4358084

[49] Yuan Y Kluiver J Koerts J de Jong D Rutgers B Abdul Razak FR. Correction. Am J Pathol. (2019) 189:479. 10.1016/j.ajpath.2018.12.002 30665558

[50] He L Ping F Fan Z Zhang C Deng M Cheng B. Salivary exosomal miR-24-3p serves as a potential detective biomarker for oral squamous cell carcinoma screening. Biomed Pharmacother. (2020) 121:109553. 10.1016/j.biopha.2019.109553 31704611

[51] Chen W Fu W Deng Q Li Y Wang K Bai Y. Multiple metals exposure and chromosome damage: exploring the mediation effects of microRNAs and their potentials in lung carcinogenesis. Environ Int. (2019) 122:291–300. 10.1016/j.envint.2018.11.020 30455104

[52] Listing H Mardin WA Wohlfromm S Mees ST Haier J. MiR-23a/-24-induced gene silencing results in mesothelial cell integration of pancreatic cancer. Br J Cancer. (2015) 112:131–9. 10.1038/bjc.2014.587 25422915 PMC4453619

[53] Jing P Xie N Zhao N Zhu X Li P Gao G. miR-24-3p/KLF8 signaling axis contributes to LUAD metastasis by regulating EMT. J Immunol Res. (2020) 2020:4036047. 10.1155/2020/4036047 32411796 PMC7204180

[54] Hao J-P Ma A. The ratio of miR-21/miR-24 as a promising diagnostic and poor prognosis biomarker in colorectal cancer. Eur Rev Med Pharmacol Sci. (2018) 22:8649–56. 10.26355/eurrev_201812_16629 30575905

[55] Yerukala Sathipati S Huang H-L Ho S-Y. Estimating survival time of patients with glioblastoma multiforme and characterization of the identified microRNA signatures. BMC Genomics. (2016) 17(Suppl. 13):1022. 10.1186/s12864-016-3321-y 28155650 PMC5260001

[56] Xiuju C Zhen W Yanchao S. SOX7 inhibits tumor progression of glioblastoma and is regulated by miRNA-24. Open Med. (2016) 11:133–7. 10.1515/med-2016-0026 28352781 PMC5329813

[57] Zhou N Yan H-L. MiR-24 promotes the proliferation and apoptosis of lung carcinoma via targeting MAPK7. Eur Rev Med Pharmacol Sci. (2018) 22:6845–52. 10.26355/eurrev_201810_16153 30402849

[58] Chen J Lou J Yang S Lou J Liao W Zhou R. MT1JP inhibits glioma progression via negative regulation of miR-24. Oncol Lett. (2020) 19:334–42. 10.3892/ol.2019.11085 31890049 PMC6933312

[59] Meng F-L Wang W Jia W-D. Diagnostic and prognostic significance of serum miR-24-3p in HBV-related hepatocellular carcinoma. Med Oncol. (2014) 31:177. 10.1007/s12032-014-0177-3 25129312

[60] Fang Z Tang J Bai Y Lin H You H Jin H. Plasma levels of microRNA-24, microRNA-320a, and microRNA-423-5p are potential biomarkers for colorectal carcinoma. J Exp Clin Cancer Res. (2015) 34:86. 10.1186/s13046-015-0198-6 26297223 PMC4546358

[61] Fredsoe J Rasmussen AKI Laursen EB Cai Y Howard KA Pedersen BG. Independent validation of a diagnostic noninvasive 3-MicroRNA ratio model (uCaP) for prostate cancer in cell-free urine. Clin Chem. (2019) 65:540–8. 10.1373/clinchem.2018.296681 30728149

[62] Rashed WM Hammad AM Saad AM Shohdy KS. MicroRNA as a diagnostic biomarker in childhood acute lymphoblastic leukemia; systematic review, meta-analysis and recommendations. Crit Rev Oncol Hematol. (2019) 136:70–8. 10.1016/j.critrevonc.2019.02.008 30878131

[63] Zhang W Fei J Yu S Shen J Zhu X Sadhukhan A. LINC01088 inhibits tumorigenesis of ovarian epithelial cells by targeting miR-24-1-5p. Sci Rep. (2018) 8:2876. 10.1038/s41598-018-21164-9 29440672 PMC5811426

[64] Zhou J Liu X Wang C Li C. The correlation analysis of miRNAs and target genes in metastasis of cervical squamous cell carcinoma. Epigenomics. (2018) 10:259–75. 10.2217/epi-2017-0104 29343084

[65] Oliveto S Alfieri R Miluzio A Scagliola A Secli RS Gasparini P. A polysome-based microRNA screen identifies miR-24-3p as a novel promigratory miRNA in mesothelioma. Cancer Res. (2018) 78:5741–53. 10.1158/0008-5472.CAN-18-0655 30072395 PMC6193539

[66] Jin F Yang R Wei Y Wang D Zhu Y Wang X. HIF-1alpha-induced miR-23a approximately 27a approximately 24 cluster promotes colorectal cancer progression via reprogramming metabolism. Cancer Lett. (2019) 440–441:211–222. 10.1016/j.canlet.2018.10.025 30393198

[67] Ehrlich L Hall C Venter J Dostal D Bernuzzi F Invernizzi P. miR-24 inhibition increases menin expression and decreases cholangiocarcinoma proliferation. Am J Pathol. (2017) 187:570–80. 10.1016/j.ajpath.2016.10.021 28087162 PMC5389363

[68] Michael JV Wurtzel JGT Mao GF Rao AK Kolpakov MA Sabri A. Platelet microparticles infiltrating solid tumors transfer miRNAs that suppress tumor growth. Blood. (2017) 130:567–80. 10.1182/blood-2016-11-751099 28500171 PMC5542851

[69] Liu J Chen Z Cui Y Wei H Zhu Z Mao F. Berberine promotes XIAP-mediated cells apoptosis by upregulation of miR-24-3p in acute lymphoblastic leukemia. Aging. (2020) 12:3298–311. 10.18632/aging.102813 32062612 PMC7066883

[70] Zhang Q Li W Liu G Tang W. MicroRNA-24 regulates the growth and chemosensitivity of the human colorectal cancer cells by targeting RNA-binding protein DND1. J BUON. (2019) 24:1476–81. 31646794

[71] Jiang W Meng K Sheng G Yang T. MicroRNA-24 inhibits the proliferation, migration and invasion and enhances chemosensitivity of human gastric cancer by targeting DND1. J BUON. (2020) 25:1001–6. 32521898

[72] Grimaldi A Zarone MR Irace C Zappavigna S Lombardi A Kawasaki H. Non-coding RNAs as a new dawn in tumor diagnosis. Semin Cell Dev Biol. (2018) 78:37–50. 10.1016/j.semcdb.2017.07.035 28765094

